# *Allium sativum* aqueous extract does not have chemo-protective effect on etoposide induced therapy-related DNA damage leading to Acute Myeloid Leukemia in albino-wistar rats

**DOI:** 10.4314/ahs.v21i2.24

**Published:** 2021-06

**Authors:** Ugochi F Ndiokwelu, Liasu A Ogunkanmi, Joseph B Minari, Ijeoma C Uzoma

**Affiliations:** 1 Molecular-Haematology Laboratory, Department of Medical Laboratory Science, Faculty of Health Sciences and Technology, College of Medicine, University of Nigeria Nsukka, Enugu Campus, Nigeria; 2 Department of Cell Biology and Genetics, Faculty of Science, University of Lagos, Akoka, Nigeria

**Keywords:** *Allium sativum* aqueous, chemo-protective effect, acute myeloid leukemia, albino-wistar rats

## Abstract

**Background:**

Therapy-related acute myeloid leukemia (t-AML) is a well-recognized clinical syndrome occurring in a significant fraction of patients who have undergone previous chemotherapy for a solid tumour.

**Objectives:**

We aim to evaluate the effect of aqueous extract of fresh Allium sativum cloves on haematological parameters, bone marrow and DNA of etoposide treated albino wistar rats. Decoction method was used to prepare plant extracts and the rats were weighed and divided into experimental and control groups. Blood and bone marrow sample were analysed and DNA fragment analysis was carried out.

**Results:**

There was progressive increase in the weight of animals that received distilled water only for the duration of the experiment while those that received etoposide only showed a sharp decrease in weight by the end of week 3. There was no significant difference in the mean of the haematological parameters in the test and control groups except for platelet count. The bone marrow smears showed no prevention of erythroblast fragmentation by the extract, in the same vein, DNA damage was not abated.

**Conclusion:**

Aqueous extract of fresh Allium sativum cloves may not be the option for the prevention of etoposide induced acute myeloid leukemia.

## Introduction

Therapy related myeloid neoplasms (t-MN) occur due to direct mutational events of chemotherapeutic agents and radiotherapy 1. Therapy-related acute myeloid leukemia/myelodysplastic syndrome (t-AML/t-MDS) is a well-recognized clinical syndrome occurring as a late complication following cytotoxic therapy 2–6 in a significant fraction of patients who have undergone previous chemotherapy for a solid tumour. The peak occurrence time of t-AML/t-MDS is 3 to 5 years after prior cytotoxic treatment, while the risk decreases markedly after the first decade^7^. Therapy-related myeloid neoplasms constitute approximately 10–20% of all cases of Acute myeloid leukemia and myelodysplastic syndrome 8. Factors associated with an increased risk of t-MN include exposure to alkylating agents, topoisomerase II inhibitors, radiation therapy 9–14 and older age at treatment, in addition to genetic susceptibility 15,16. Also, t-MN after anthracyclines and/or topoisomerase II inhibitors are associated with occurrence of AML- myeloid/lymphoid leukemia (MLL) translocation at 11q23. Traditional herbal medicine could play pivotal roles in cancer treatment 17. *Allium sativum*, commonly known as garlic, has been used as therapeutic medicinal agent for thousands of years to treat a variety of diseases such as cancer and cardiovascular diseases 18. Therefore, we evaluated the effect of aqueous extract of fresh Allium sativum cloves on the haematological parameters, bone marrow and gel pattern of genomic DNA extracted from etoposide treated albino rats.

## Methods

### Chemical used

Etoposide VP-16 obtained from L'PaceMaker Pharmaceuticals Limited was used for the study and all other reagents were prepared in the laboratory.

### Laboratory animals used

A total of 25 adult albino rats with average weight of 96–136g were used and were obtained from the University of Lagos College of Medicine animal house. They were housed in standard clean rat cages at room temperature (18–25°C) and fed with standard pellet rat chow (Korede Farms, Lagos-Nigeria) and tap water *ad libitum*. They were maintained under uniform conditions of natural photoperiod (12 hours' light-dark cycle) and humidity (61–95%).

### Plant used

The garlic bulbs used were purchased from Oyingbo market, Lagos state. Its cloves were identified and authenticated taxonomically at the Herbarium Unit of Botany Department of the University of Lagos and a voucher specimen number (LUH 7338) was deposited.

### Plant extract preparation

Decoction method was used to prepare the plant extract. The garlic cloves were peeled, washed and blended using electronic blender (Freepour) and 1.2kg of the ground garlic was put into a 5000 mL conical flask and 3 L of distilled water was poured in and mixed with ground garlic. The sample was then placed on a regulated water mantle and heated at 70°C for 15mins. The heated preparation was then filtered using a 250 mm mesh followed by Whatman filter paper, 55 mm in diameter (Whatman Inc) and the residue taken through the same boiling process again. The combined filtrate was then concentrated in a water bath at 50°C leaving a paste-like garlic extract. Phytochemical screening employing standard procedure^18^ was carried out. Afterwards, stock solution of 10 g/100 mL was prepared and stored in a refrigerator at 4°C till use 19.

### Experimental design

At the commencement of the experiment, the rats were weighed and classified into 5 groups, of 5 animals each. The classification was as follows;

Group RP: (Positive control) - Rats that received distilled water only

Group RN: (Negative control) – Rats that received 6 mg/kg bw. /week of etoposide VP-16

Group RA: Rats that received 6mg/kg bw. /week of etoposide VP-16 and 100 mg/kg bw. /day of extract

Group RB: Rats that received 6 mg/kg bw. /week of etoposide VP-16 and 200 mg/kg bw. /day of extract

Group RC: Rats that received 6 mg/kg bw. /week of etoposide VP-16 and 300 mg/kg bw. /day of extract

Induction was done by intraperitoneal injection of calculated doses (according to average body weight) of etoposide VP-16 (6 mg/kg bw. /week). This was administered to the 3 experimental groups and the negative control group.

Prepared aqueous *Allium sativum* extract was administered simultaneously following etoposide induction. Three different concentrations of extract (100 mg/kg bw. /day, 200 mg/kg bw. /day and 300 mg/kg bw. /day) were prepared with respect to LD50 results as documented by Chris-Ozoko^19^. Each experimental group received one of the three concentrations by gavage using a cannula every day for 3 weeks. The positive control group (RP) received distilled water only.

### Experimental site and Ethical Statement

The work was carried out in the animal house (Botanical garden) of the University of Lagos in accordance with the code of ethics of the World Medical Association (Declaration of Helsinki, 2008) for animal experiment with consent from the University of Nigeria Teaching Hospital, Health Research Ethics Committee (NHREC/05/01/2008B-FWA00002458-IRB00002323) guidelines for experiment with whole animals.

### Complete blood count and bone marrow analysis

At the end of 3 weeks, 2 mL of blood was collected from the eye vein of the animals in each group and analysed for complete blood count (CBC) using a fully automated 3-part haematology analyser (Alfa Swelab, Sweden). Afterwards, the animals were anaesthetized by inhalation of isoflurane and then euthanized by cervical dislocation. The femurs of the animals were then carefully removed and cleaned, the epiphyseal ends were cut off and solid bone marrow flushed unto a petridish using 0.5 mL saline, needle and syringe. Marrow suspension was centrifuged, and sediment used to prepare slides which were fixed in absolute methanol and stained with Wright's stain. The stained slides were then examined microscopically under 100x magnification with immersion oil.

### DNA extraction and electrophoresis

Genomic DNA was also extracted from blood using lysis buffer, chloroform and cold ethanol then suspended in Tris-EDTA buffer and gel electrophoresis of extracted DNA was done using EDVOTEK M12 horizontal electrophoresis apparatus.

### Data analysis

All statistical analysis were performed using Graphpad prism version 7.02. Two-way analysis of variance (ANOVA) followed by Tukey's Multiple comparison test to determine which experimental group showed the most improvement in blood haematological parameters and least decrement in weights was calculated. Results were expressed as mean ±SD and values of p < 0.05 were considered statistically significant.

## Results

Results obtained from the qualitative phytochemical screening of aqueous Allium sativum extract are shown in Table 1. The phytochemical screening revealed the presence of saponins, flavonoids, tannins, alkaloids, molisch, proteins, reducing sugars and steroids with an acidic pH. Anthraquinolones and terpenoids were absent.

**Table 1:** Qualitative phytochemical screening of aqueous extract of *Allium sativum* cloves

S. No.	Phytochemical	Indication in Aqueous Extract
1	Saponins	(+)
2	Flavonoids	(+)
3	Tannins	(+)
4	Alkaloids	(+)
5	Moilsch	(+)
6	Reducing sugars	(+)
7	Terpenoids	(−)
8	Proteins	(+)
9	Steroids	(+)

**Key:** (+) Presence of phytochemical(s)

(−) Absence of phytochemical(s)

Table 2 showed significant decrease (p<0.05) in weights of rats with simultaneous administration of etoposide and extract across the experimental groups with each new week.

**Table 2:** Effect of aqueous extract of Allium sativum cloves on average weight of etoposide treated rats

Weeks	RP(g)	RN(g)	RA(g)	RB(g)	RC(g)
**Week 1**	87.00 ± 2.9	138 ± 8.3	117.00 ± 2.8	125.80 ± 5.1	140.00 ± 6.0
**Week 2**	96.20 ± 3.5	141.75 ± 7.3	115.66 ± 2.9	114.50 ±10.0	136.75 ± 7.3
**Week 3**	109.40 ± 4.6	116.66 ±18.5	≠	108.00 ± 7.5	133.3 ± 8.0

**Key:** ≠- All Dead, values are means of 3 replicates ± SD

**Plate 1:**
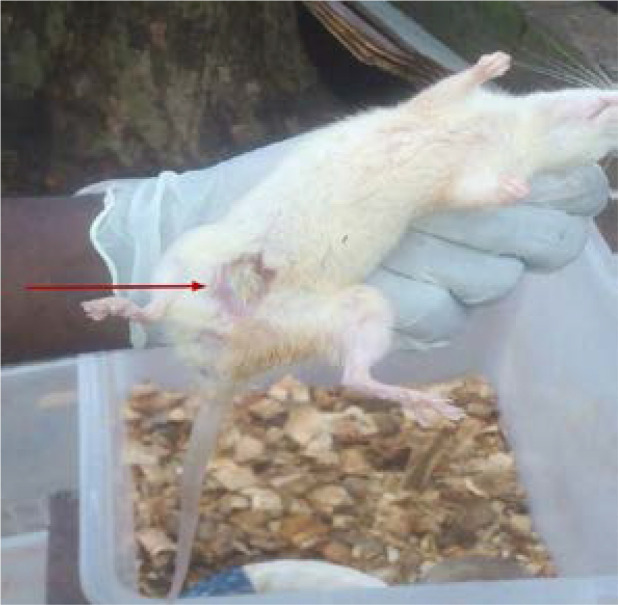
Etoposide induced-alopecia on peritoneal region of rats treated with Etoposide only. Arrow indicates the location of etoposide induced alopecia.

**Figure 1:**
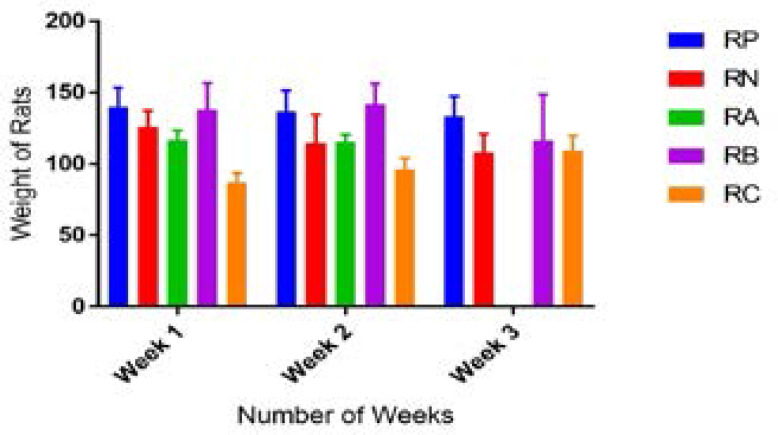
Effects of aqueous extract of *Allium sativum* cloves on weight trend of etoposide treated rats for 3 weeks Legend: RP: (Positive control) - Rats that received distilled water only RN: (Negative control) – Rats that received 6mg/kg bw./week of etoposide VP-16 RA: Rats that received 6mg/kg bw./week of etoposide VP-16 and 100mg/kg bw./day of extract RB: Rats that received 6mg/kg bw./week of etoposide VP-16 and 200mg/kg bw./day of extract RC: Rats that received 6mg/kg bw./week of etoposide VP-16 and 300mg/kg bw./day of extract

Post week one examination of experimental animals following administration of etoposide showed localized alopecia shown in Plate 1, which was generalized by the end of week two. There was a level of constancy in the average weight of animals that received distilled water only (RP) for the duration of the experiment as shown in Figure 1. Rats that received etoposide only (RN) showed a progressive decrease in average weight throughout the weeks. The experimental groups RA & RB showed gradual decrease in average weight at the end of each week of administration while RC showed a progressive increase in average weight in the 3 weeks.

**Figure 2:**
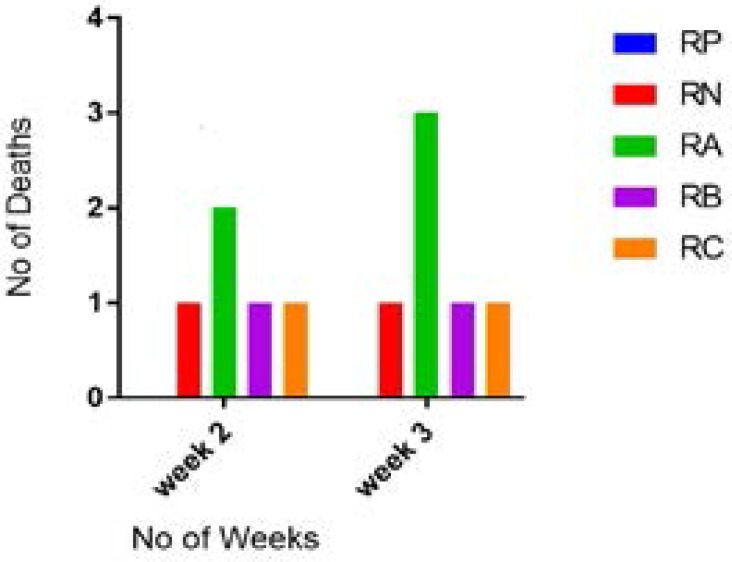
Effects of aqueous extract of *Allium sativum* cloves on survival rate of etoposide treated rats. Legend: RP: (Positive control) - Rats that received distilled water only RN: (Negative control) – Rats that received 6mg/kg bw./week of etoposide VP-16 RA: Rats that received 6mg/kg bw./week of etoposide VP-16 and 100mg/kg bw./day of extract RB: Rats that received 6mg/kg bw./week of etoposide VP-16 and 200mg/kg bw./day of extract RC: Rats that received 6mg/kg bw./week of etoposide VP-16 and 300mg/kg bw./day of extract

**Plate 2:**
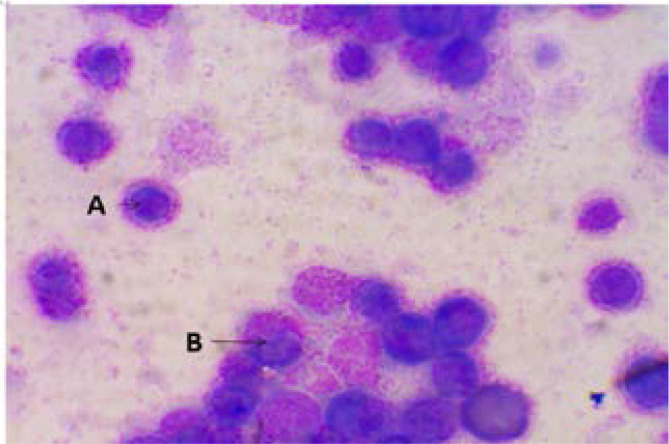
Bone marrow smear stained with Wright's stain from group RP (X100). RP: Rats administered with distilled water only. A. Basophilic Erythroblast. B. Megakaryocyte with normal nuclear budding. Megakaryopoiesis is active with normal nuclear budding. Myelopoiesis is active as well as erythropoiesis, basophilic erythroblast (shown with the arrow) appear to have normal morphology.

**Plate 3:**
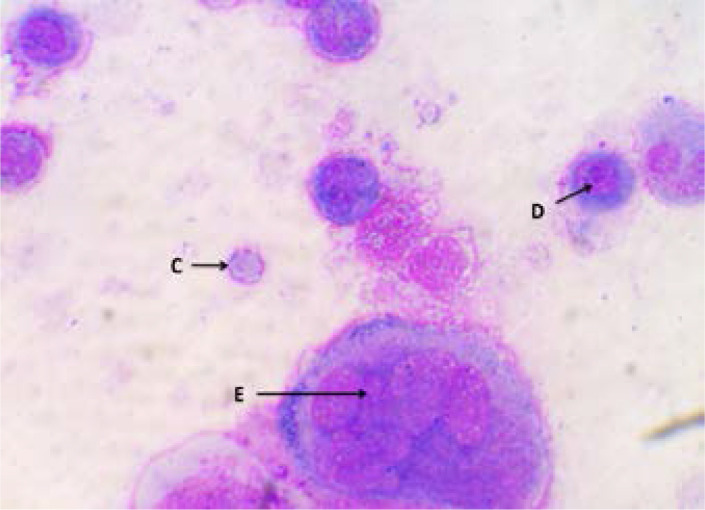
Bone marrow smear from RN showing erythroblast with micronuclear fragmentation and highly active megakaryocyte (Wright's stain X100). RN: Rats that received 6 mg/week of etoposide VP-16. C. Erythroblast with micro-nuclear fragmentation. D. Small lymphocytes. E. Highly active megakaryocyte.

**Plate 4:**
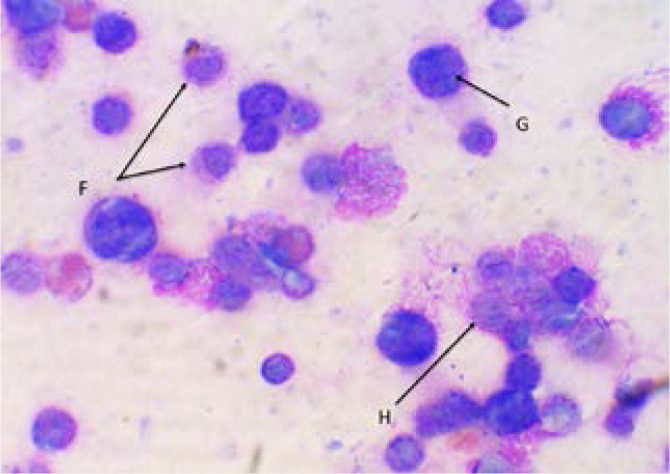
Bone marrow smear from RB (Wright's stain X00). RB: Rats that received 6 mg/kg bw./week of etoposide VP-16 and 200 mg/kg bw./day of extract. F. Myeloblasts. G. Erythroblast. H. Erythroblast with micronuclear fragmentation

**Plate 5:**
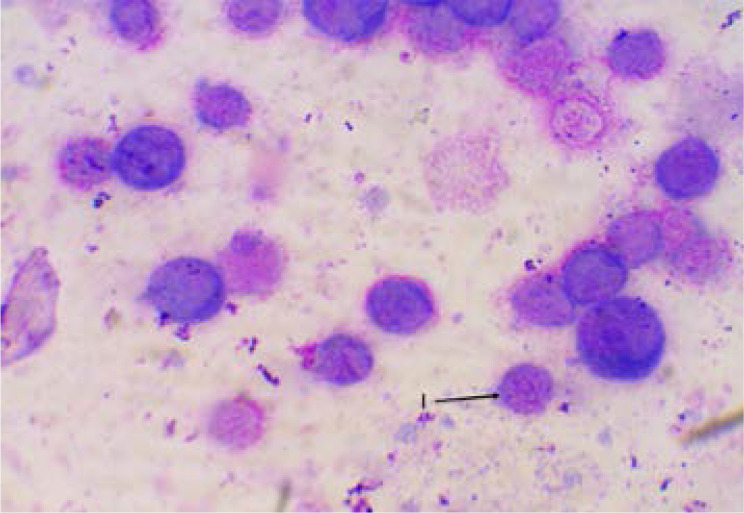
Bone marrow smear from RC showing myeloblast with undefined nuclear outline. RC: Rats that received 6 mg/kg bw./week of etoposide VP-16 and 300 mg/kg bw./day of extract. I. Myeloblast with undefined nuclear outline.

Figure 2 showed zero deaths at the end of week one and at least two deaths recorded in each group at the end of week three. Plate 2 shows the result of the haematological examination of stained bone marrow smear from group RP. Haematological examination of smear seen in Plates 3 and 4 respectively revealed actively dividing megakaryocytes with normal nuclear budding, normal and active myeloid stem cells, erythroblasts with micronuclear fragmentation in rats from groups RN and RB. Examination of smear from group RC is seen in Plate 5 and shows myeloblasts with undefined nuclear cytoplasm.

There was no significant (p>0.05) difference observed in the complete blood count parameters (Table 3) amongst the treated groups in comparison with the positive control.

**Table 3:** Effects of aqueous extract of *Allium sativum* cloves on haematological parameters of etoposide treated rats

Parameters	RP	RN	RB	RC
**RBC (x10^6^/mm^2^**	5.65 ± 0.75	0.36 ± 0.12	4.8 ± 0.86	4.2 ± 0.04
**PCV (%)**	28 ± 3.70	23.4 ± 6.45	31.1 ± 3.65	26.9 ± 2.55
**Hb (g/dl)**	9.9 ± 3.50	8.85 ± 3.05	10.8 ± 1.45	10.3 ± 0.85
**MCV (%)**	64.2 ± 3.60	64.80 ± 5.00	60.2 ± 0.90	62.9 ± 5.05
**WBC (x 10^3^/mm3)**	5.8 ± 1.70	6.1 ± 1.70	6.2 ± 0.45	4.4 ± 0.65
**PLT (x10^3^/mm^3^)**	484.9 ± 61.50*	595 ± 85.0	615.2 ± 85.00	635.5 ± 185.00*
**Lymphocytes (%)**	76.8 ± 9.65	71.9 ± 9.05	81.3 ± 0.05	79.4 ± 4.05
**Granulocytes (%)**	16.7 ± 7.05	20.1 ± 12.30	9.7 ± 1.90	12.5 ± 2.10

**Key:** RBC = Red blood cells, PCV = Packed cell volume, WBC = White blood cells, MCV = Mean cell volume, HB = Haemoglobin

Values are means of 3 replicates ± SD

* significant change

## Discussion

The evidence of erythroblast nuclear fragmentation and presence of myeloid with undefined nuclear outline seen in bone marrow smear of negative control group (RN) and treatment group (RC) was suggestive of possible early manifestations of DNA damage on the erythroid cell precursors due to etoposide administration. There were also fewer myeloid and erythroid precursor cells in the treatment groups when compared with the positive control group (RP). This was corroborated by the study done by Papiez 24, reporting an initial short-term substantial decrease in the percentage of myeloid precursors and erythroid nucleated cells in bone marrow smear of rats caused by etoposide administration.

There was no significant difference between the complete blood count parameters amongst the treated groups in comparison with the positive control. This could be due to the fact that the evidence of manifestations of clinical and diagnostic symptoms of etoposide induced DNA damage leading to leukemia which is seen as abnormal blood cell counts, particularly the WBCs, develop about 2–3 years post exposure to the chemotherapeutic agent and is also largely dependent on long exposure to the drug even at sub-lethal doses 25. Both conditions however were not covered in this experiment. The significant increase in the platelet count of the treated groups (RB) and RC with the most increase seen in the group that received the highest dose of extract ie 300mg/kg bw. /day (RC) as compared to the positive control group (RP), was in contrast with Olaniyan et al,^26^ who reported a non-significant change in platelet count amongst adult wistar rats treated with aqueous *Allium sativum* extract. This significant increase in platelet count with increase in the concentration of the extract administered may have been as a result of the combined action of etoposide and increased extract administration in this group.The slight deviation from normal displayed by bands obtained from agarose gel electrophoresis particularly with RN2 may have arisen from DNA damage caused by etoposide administration. This is in line with the report done by Berger et al^27^, who proposed that the imprecise repair of etoposide induced double stranded breaks through non-homologous DNA end-joining (NHEJ), results in chromosomal translocations involving cellular oncogene which induces the leukomogenic pathway a few years post exposure to the drug. The RB1 and RC1 however displayed a slight similarity with RN2 suggesting that the extract administered may actually not have prevented the damage done by etoposide. This may be due to the reported mechanism of action of Allium sativum extracts. In the study done by Lamm and Riggs 21, it was reported that Allium sativum metabolites induce apoptosis in leukemic cells through the mitochondria-dependent caspase cascade which causes a significant reduction of anti-apoptotic Bcl-2 that results in the release of cytochrome C and the activation of caspase-3 28. The proprietary aging process produces an odourless preparation and converts the harsh, unstable organosulphur compounds in garlic (e.g allicin) into milder and more beneficial compounds which are responsible for the anticancer effects of *Allium sativum* 29. Johnson and Yedjou^20^, reported a free radical activity and a direct cytotoxic effect on cancer cells particularly leukemic cell lines. The anti-leukaemic properties of *Allium sativum* have been attributed to its sulphur containing metabolic by-products which are formed on crushing and long standing from its major phytochemical allicin. Allicin and its metabolites reportedly induce apoptosis in leukemic cells through the mitochondria-dependent caspase cascade which causes a significant reduction of the anti-apoptotic Bcl-2 that results in the release of cytochrome C and activation of caspase-3 21.

## Conclusion

The study showed that aqueous extract of fresh *Allium sativum* cloves may not be the option for the prevention of etoposide induced leukemia in rats since the bone marrow smears showed that the plant extract did not prevent erythroblast fragmentation which may be indicative of existing DNA damage following etoposide treatment.

## Figures and Tables

**Plate 6:**
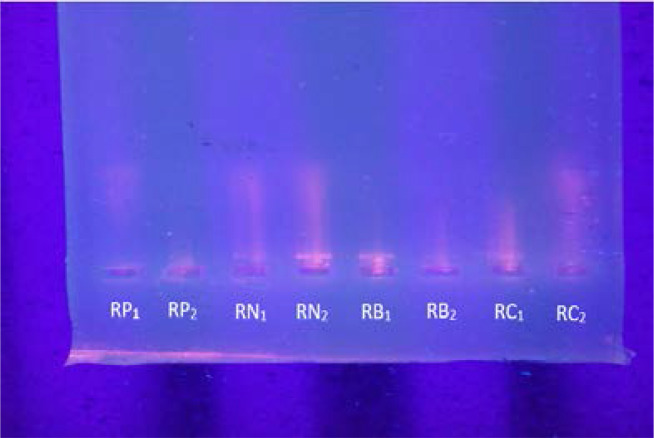
Agarose gel electrophoresis bands of extracted DNA from blood of rats. It shows slight variation in gel pattern of DNA from RN, RB and RC. Legend: Plate 6 shows electrophoretic bands of DNAs obtained from blood cells of rats RP: (Positive control) - Rats that received distilled water only RN: (Negative control) – Rats that received 6 mg/kg bw./week of etoposide VP-16 RA: Rats that received 6 mg/kg bw./week of etoposide VP-16 and 100 mg/kg bw./day of extract RB: Rats that received 6 mg/kg bw./week of etoposide VP-16 and 200 mg/kg bw./day of extract RC: Rats that received 6 mg/kg bw./week of etoposide VP-16 and 300 mg/kg bw./day of extract
